# Malaria vectors of Timor-Leste

**DOI:** 10.1186/1475-2875-9-40

**Published:** 2010-02-02

**Authors:** Robert D Cooper, Michael D Edstein, Stephen P Frances, Nigel W Beebe

**Affiliations:** 1 Australian Army Malaria Institute, Gallipoli Barracks, Enoggera, Queensland, Australia; 2 School of Biological Sciences, University of Queensland, Goddard Building, St Lucia, QLD 4072, Australia; 3 CSIRO Entomology, Long Pocket Laboratories, Indooroopilly, QLD 4068, Australia

## Abstract

**Background:**

The island of Timor lies at the south-eastern edge of Indonesia on the boundary of the Oriental and Australian faunal regions. The country of Timor-Leste, which occupies the eastern part of the island, is malarious but anopheline faunal surveys and malaria vector incrimination date back to the 1960 s. Over the last decade the malaria vectors of south-east Asia and the south-west Pacific have been intensely studied using molecular techniques that can confirm identification within complexes of isomorphic species. The aim of this study is to accurately identify the *Anopheles *fauna of Timor-Leste using these techniques.

**Methods:**

The survey was carried out over the period February to June 2001. Standard entomological techniques - human landing collections, larval collections and CO2 baited light traps - were used to collect anophelines from the main geographical regions: coastal plains, inland plains, and highlands. Specimens were processed for identification by morphology and genotyped for the ribosomal DNA ITS2 by restriction analysis and/or DNA sequencing. Phylogenetic relationship of *Anopheles sundaicus *and *Anopheles subpictus *individuals was also assessed using DNA sequences from the ITS2 and mitochondrial cytochrome-b. All specimens, other than those from larval surveys, were processed to detect the presence of the *Plasmodium *parasite circumsporozoite protein by ELISA for vector incrimination.

**Results:**

Of 2,030 specimens collected, seven species were identified by morphology: *Anopheles barbirostris, Anopheles aconitus, Anopheles annularis, Anopheles maculatus, Anopheles peditaeniatus, An. sundaicus *and *Anopheles vagus*. These were confirmed by molecular analysis with the addition of *Anopheles flavirostris *and an unidentified species designated here as *An. vagus *genotype B. This latter species was morphologically similar to *An. vagus *and *An. subpictus *and is likely to be the *An. subpictus *described by other workers for Timor. However, genetically this species showed strong affinities to the *An. sundaicus *complex. *Anopheles vagus *was the most common species but was rarely collected coming to bite humans; *An. barbirostris *and *An. vagus *genotype B were the two most common species collected in human landing catches and both were found positive for CS protein.

**Conclusions:**

The anopheline fauna of Timor-Leste is of Oriental origin with no evidence of elements from the Australian Region. The existence of species complexes will make the use of morphological markers problematic in the country. Using molecular analysis a number of issues regarding the anopheline fauna of Timor-Leste were resolved and nine putative species of *Anopheles *were identified; two species: *An. barbirostris *and *An. vagus *genotype B, were incriminated as malaria vectors.

## Background

Timor, of which Timor-Leste (formerly East Timor) is part, is the largest island at the eastern end of the Lesser Sunda Islands which form part of the Malayan Archipelago and the country of Indonesia from which Timor-Leste recently gained independence. Malaria is endemic in Timor-Leste, though transmission is not high, tending to be mesoendemic on the coast, hypoendemic in the inland lowland regions, and very low to absent in areas over 500 m above sea level (asl) [1]. Malaria transmission peaks at the end of the wet season, which occurs from November to May [2].

The island of Timor lies on the border of the Oriental and Australian faunal regions, and while, like Australia, it is of Gondwanic origin the island has never been connected with Australia, even during the glacia-maxima of the Pleistocene era and the existence of the Shula Shelf [3]. Its *Anopheles *fauna appears to be distinctly Oriental, although it has been reported that members of the Punctulatus Group, which includes the major malaria vectors of the south-west Pacific, may occur there [4]. Various publications have listed the anopheline fauna of Timor-Leste, however they tend to be general in nature covering either the whole of Indonesia or south-east Asia [5,6]. Faunal surveys by Portuguese workers mentioned 11 species [7,8]. In 1975, Lien and colleagues provided a list of 14 anopheline species for Indonesian Timor [9]. Four species have been incriminated as vectors of malaria: *Anopheles barbirostris, Anopheles sundaicus, Anopheles subpictus *and *Anopheles aconitus*. Of these *An. barbirostris, An. subpictus *and *An. sundaicus *are considered as the main vectors [7-9].

The discrepancies presented by the various authors with regards to anopheline speciation in Timor-Leste highlights the problems of using alpha taxonomy for identifying specimens especially where cryptic or isomorphic species are involved. Here the problem is exacerbated as Oriental species have, over the last 5 M years, moved down from the south-east Asian mainland through the islands of the Malayan Archipelago allowing for founder effects and island isolationism, resulting in cryptic species with subtle changes in morphology.

Accurate species identification, which allows important vectorial parameters to be applied to species, is crucial in malaria transmission studies and in implementing and monitoring control strategies. Over the last ten years a considerable amount of work has been done to validate the identity of the malaria vectors of south-east Asia and the south-west Pacific using molecular techniques. Species identification is now predominantly based on a polymerase chain reaction (PCR) of the ribosomal DNA (rDNA) internal transcribed spacer 2 (ITS2) region either through restriction fragment length polymorphism analysis (RFLP) or an allele-specific PCR (AS-PCR) [10,11]. To date, these techniques have not been applied to resolve the species identity of the malaria vectors in Timor-Leste; this study rectifies this.

In support of Timor-Leste's transition to independence, a United Nations Peace Keeping Force was deployed in September 1999. During the first six months the Australian Defence Force (ADF) contingent suffered 267 malaria infections (4.8% of 5,500 personnel) consisting of 43 cases of *Plasmodium falciparum *and 21 cases of *Plasmodium vivax *in-country and 212 cases of relapsing *P. vivax *on return to Australia [12]. During the period October 2000 to June 2001 anophelines surveys were conducted on the western border of Timor-Leste to monitor the risk of malaria to ADF personnel deployed there as part of the UN Peace Keeping Force. These collections were made during the wet season when it would be expected that both vector densities and malaria transmission would be heightened. The material collected in this study was analysed by PCR to provide accurate species identification and by ELISA to identify the infectious stage of the *Plasmodium *parasite and incriminate malaria vectors.

## Methods

### Climate and geography

The island of Timor lies 9°S and 125°E; Timor-Leste occupies 18,900 sq km (about two-thirds) of the eastern part of the island. The climate is tropical monsoon with distinct wet and dry seasons. Precipitation ranges between 1,000-2,000 mm p.a.; the wet season occurs from November to May and accounts for 80-85% of the annual rainfall, whereas the dry season is from June to October. The country is mountainous with a central range up to 2,900 m asl separating the north and south coasts. There are three main geographical regions: a narrow coastal plain (0-20 m asl), inland lowland plains (100-500 m asl), and highland regions (>500 m asl).

### Collection methods

**Figure 1:**
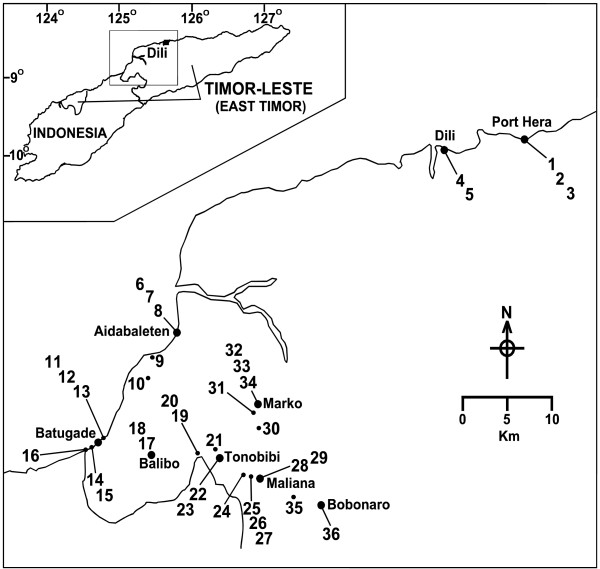
**Map of the survey region indicating collection locations and main towns**.

Collections of anophelines were made from the Dili area (coastal) and the Bobonaro District including coastal, lowland inland and highland areas (Figure 1). Human landing collections were made over the period February to June 2001. Sunset was approx 1915 hr and sunrise 0645 hr. Three human landing catches (HLC) (1900-0700 hr) were conducted, one on the coast at the town of Batugade and two inland, around the towns of Tonobibi and Marko. Forty-four HLC (1900-0100 hr) were subsequently made, twenty-two of which were on the coast around the towns of Batugade, Aidabaletan and Port Hera (all <10 m asl); sixteen in the inland lowland region around the towns of Tonobibi (170 m asl), Maliana (240 m asl) and Marko (100 m asl); six in the highlands with four at Balibo (530 m asl), and two at Bobonaro (900 m asl). The purpose of these HLC was to determine which species were present in the different geographical zones, which species were biting humans, and to provide specimens for circumsporozoite (CS) protein determination. Adult collections were also made using CO_2 _baited EVS traps [13]. Seventeen traps were set: six coastal, eight in the inland low land areas and three in the highland town of Balibo. Larval surveys were also conducted throughout the Bobonaro District and around Dili and Port Hera over the period October 2000 to July 2001. All specimens were stored frozen at -20°C in the field and transported on dry ice back to the Army Malaria Institute, Brisbane, for further analysis.

### Species identification

All larvae were reared to adults in the site water from which they were collected. All adult females were identified morphologically using the keys of Bonne-Wepster and Swellengrebel, Reid, and O'Connor and Soepanto [6,14,15]. Species identified were further characterised by PCR-RFLP studies of the rDNA ITS2 which assesses crude sequence variation and is augmented by the presence of indels (insertions and deletions) common in this region that can produce diagnostic band profiles in related species [11,16]. Following PCR amplification, products were digested with restriction enzymes (*Hinf *I, *Hha *I, *Tru *9I, *Hsp *92 III, *Ali *I, *Sau *3AI, *Sal *I, *Msp *I, *Dde *I, and *Rsa *I) that recognise four nucleotide motives to produce diagnostic RFLPs of the ITS2 region.

### DNA sequencing and analyses

The rDNA ITS2 region was sequenced from species belonging to recognised complexes based on differential PCR-RFLP profiles [17]. The mtDNA cytochrome oxidase (cyt-b) fragment was amplified using both PCR and sequencing primers CBsunA and CBsunB [18]. The 25 μl PCR reaction contained 1.75 mM MgCl_2_, 200 pM of each dNTP, 0.4 μM of each primer, 0.5U of *Taq *DNA polymerase (Fisher Biotech, WA, Australia) and approximately 1-20 ng of genomic DNA template (1 μl of extraction). Cycling conditions included initial denaturing at 93°C for 4 min followed by 35 cycles of 93°C for 1 min, 50°C for 1 min, 72°C for 1.5 min. The PCR product was purified using a QIAquick PCR purification kit (Qiagen) and sequenced by the Australian Genome Research Facility (University of Queensland, St Lucia, Australia) on an ABI3730*xl*.

Additionally, ITS2 and cyt-b sequences were obtained from *An. subpictus *specimens collected from Papua New Guinea (PNG) and Vietnam. *Anopheles sundaicus *and *An. subpictus *ITS2 and cyt-b sequences were obtained from Genbank using both species' name searches and nucleotide Blast (Blastn) searches through the National Centre for Biotechnology Information (NCBI). Sequences drawn from GenBank were limited to *An. sundaicus *and related *An. subpictus *species (Table 1). Sequence alignments for both markers were generated in ClustalW [19] and edited for length for ITS2 and cyt-b and length and repeat structure (ITS2 only). Sequence alignments then underwent Maximum Likelihood analyses using the GTR+Γ+I model in PhyML 2.4.4 [20]. Branch support was evaluated by the bootstrapping method with 100 replicates in PhyML.

**Table 1:** ITS2 and mitochondrial DNA cyt-b sequences used in the phylogenetic study of *An. sundaicus, An. subpictus*, and *An. vagus *genotype B.

Species	Site	ITS2 accession number*	Cyt-b accession number
*An. sundaicus*- TL415	Timor-Leste	GQ480825	GQ480835
*An. sundaicus*- TL417	Timor-Leste	GQ480826	GQ480836
*An. sundaicus*-I		AY768540	
*An. sundaicus*-II		AY768541	
*An. sundaicus*-III		AY768542	
*An. sundaicus*-IV	Malaysia/Borneo	AY768543	
*An.vagus*B-411	Timor-Leste	GQ480824	GQ480833
*An. vagus *B-412	Timor-Leste	GQ480823	GQ480834.1
*An. subpictus*-Viet207	Vietnam	GQ480828	GQ480830
*An. subpictus*-Viet206**	Vietnam		GQ480829
*An. subpictus*PNG170	PNG	GQ480827	GQ480831
*An. subpictus*PNG100**	PNG		GQ480832
*An. subpictus*-SL1	Sri Lanka	AY406619	
*An. subpictus*-SL2	Sri Lanka	AY049004	
*An. subpictus*-SL3	Sri Lanka	AY406615	
*An. subpictus*-SL4	Sri Lanka	AY406616	
*An. subpictus*-SL5	Sri Lanka	AY406613	
*An. subpictus*-India1	India	EF601869	
*An. subpictus*-India2	India	EF601868	
*An. subpictus*-India3	India	EF601870	
*An. sundaicus*-KK19	Vietnam		AY672293
*'An. sundaicus*-VBL92	Vietnam		AY672286
*An. sundaicus*-TP26	Vietnam		AY672299
*An. sundaicus*-VBL93	Vietnam		AY672287
*An. sundaicus*-VHCM59	Vietnam		AY672288
*An. sundaicus*-KK57	Vietnam		AY672295
*An. sundaicus*-VHCM24	Vietnam		AY672290
*An. sundaicus*-MAT3	Vietnam		AY672310
*An. sundaicus*-TP22	Vietnam		AY672301
*An. sundaicus*-TP19	Vietnam		AY672304
*An. sundaicus*-TPG6	Vietnam		AY672299
*An. sundaicus*-TP25	Vietnam		AY672305
*An. sundaicus*-INA7343	Sumatra Indonesia		AY672338
*An. sundaicus*-INL12	Sumatra Indonesia		AY672322
*An. sundaicus*-INL2	Sumatra Indonesia		AY672318
*An. sundaicus*-INL10	Sumatra Indonesia		AY672321
An. sundaicus-MAG4	Malaysia/Borneo		AY672316
*An. sundaicus*-MAG3	Malaysia/Borneo		AY672315
*An. sundaicus*-MAG2	Malaysia/Borneo		AY672314

*Anopheles sundaicus ITS2 Variant I is identical to *An. epiroticus*; Variants I, II and III appear as copy polymorphisms within individuals from Sumatra and Java in Indonesia.

**ITS2 sequence is identical to the one above.

### Sporozoite detection

All adult mosquitoes collected in human landing catches were processed for the presence of CS protein of *P. falciparum *and *P. vivax *210 (and 247 variant) using a rapid dip stick method (VecTest™ - Medical Analysis Systems Inc, Camarillo, California, USA) [21]. Positive samples were confirmed using the standard ELISA methods of Dr Robert Wirtz (Centers for Disease Control and Prevention, MS F42, Atlanta, GA 30341-3717, USA). CS protein-positive specimens were scored as those with absorption values greater than twice the mean (*n *= 5) negative control value [22]. Only the head and prothorax were used in this method; the remainder of the specimen was kept for molecular analysis.

## Results

### Collections and species composition

Three HLC were conducted 1900-0700 hr and indicated that while anophelines fed sporadically throughout the night, there was a peak feeding time in the first hour of the night when the majority of feeding occurred. Accordingly the remaining HLC were made from 1900-0100 hr. Thirty-eight larval collections were made throughout the survey area. Figure 1 shows the locations where adult, larval and trap collections were made.

A total of 2030 anophelines were collected, 932 from HLC, 1082 as larvae, and 16 from trap collections. From these the following seven species were identified by morphology: *An. barbirostris, An. peditaeniatus, An. aconitus, An. annularis, An. maculatus, An. sundaicus *and *An. vagus*. All species, except *An. maculatus*, were collected in HLC. *Anopheles barbirostris, An. vagus, An. maculatus *and *An. annularis *were also collected as larvae. The results of CO_2 _baited traps collections were poor and from the 17 traps set only 16 anophelines were collected: *An. vagus *(×12), *An. aconitus *(×1), *An. annularis *(×2) and *An. peditaeniatus *(×1).

Collections from highland sites were limited, HLC (1900-0100 hr) resulted in one *An. vagus *from Balibo and no anophelines were collected using this method in the vicinity of Bobonaro. Around Balibo and Bobonaro the terrain is rugged and steep with little flat ground and few potential anopheline larval habitats. Only two *An. vagus *larval sites were located at Balibo and one at Bobonaro. Table 2 shows the types of collections and the numbers of each species collected; the site numbers match those in the Figure 1.

**Table 2:** *Anopheles *species and numbers collected, their location and method of collection in the Bobonaro District and Dili area of Timor-Leste during the period February to June 2001

Species	No. collected	Site Nos. (as shown in Figure 1.)	**Type of collection**^**1**^
*An. barbirostris*	395	1, 3, 8, 10, 13, 14, 19, 20, 22, 32	L, HLC
*An. peditaeniatus*	66	1, 22, 28, 32	HLC, LT
*An. aconitus*	14	1, 14, 20, 32	HLC, LT
*An. annularis*	28	20, 22, 26, 27, 32	L, HLC, LT
An. maculatus	176	19, 25, 26, 30, 31, 35,	L
*An. flavirostris*	4	22, 32	HLC
*An. sundaicus*	8	1	HLC
*An. vagus*	901	1, 2, 3, 6, 7, 8, 9, 10, 11, 12, 13, 15, 16, 17, 18, 19, 20, 21, 22, 23, 24, 25, 26, 27, 28, 29, 30, 31, 32, 33, 34, 36	L, HLC, LT
*An. vagus *genotype B	414	1, 4, 5, 6, 8, 9, 14, 22	L, HLC

^1 ^L = larval collection, HLC = human landing catches, LT = CO_2 _baited light trap

### Species collected

*Anopheles barbirostris *was common and widespread throughout the coastal and inland lowland regions. It was one of the dominant anophelines collected in HLC both on the coast (31.2% of total collection) and inland (38.2% of total collection) though only three larval sites were located. These sites were large permanent bodies of water with well-established flora and fauna. Sixty-six specimens were analysed by PCR-RFLP and there appeared to be only one ITS2 genotype.

*Anopheles peditaeniatus *was collected in HLC, but not as larvae. This species was rarely found on the coast (1/66) but was common in the inland lowland plains (65/66). The ITS2 region was examined in 29 specimens; all were found to be the same RFLP genotype. It was determined that the restriction enzyme *Dde *I would produce RFLPs that would reliably separate *An. sinensis, An. crawfordi *and *An. peditaeniatus*. This enzyme identified all the Timor-Leste material as *An. peditaeniatus *(confirmed by sequencing: GenBank accession number AF543862). Additionally sequences from the ITS2 region from these specimens matched those of *An. peditaeniatus *from China [23]. With many of the specimens collected, identification based on morphological characters was difficult, and even using all the taxonomic keys, separation from *Anopheles nigerrimus *and *Anopheles argyropus *was not always possible.

*Anopheles aconitus *was collected on the coast and inland (<500 m asl); all in HLC with no larval sites found. Of the 26 specimens collected 18 were subjected to molecular analyses [16]. Of these 14 were *An. aconitus *and four were *An. minimus*. All 18 specimens were originally identified as *An. aconitus *based on the pale scaling on the proboscis. Given that this is an unusual character for *Anopheles minimus*, the four specimens of this type were sequenced and found to be *Anopheles flavirostris. Anopheles aconitus *was found coastally and in the inland lowland plains; An. flavirostris was only found in the inland plains. The ITS2 sequence from *An. aconitus *(GQ500119) was unique although it shows high similarity (97-98%) to *An. aconitus *isolates from south-east Asia, whereas *An. flavirostris *(GU062188) showed one nucleotide difference to the same species from the Philippines.

*Anopheles annularis *was collected as larvae and in HLC; all collections were made from the inland plains. The numbers collected in HLC were low; larval habitats were natural ground pools with established flora and fauna. All specimens showed the same ITS2-RFLP profiles and a subset were sequenced (GU062187) and showed 99% identity with the same species from south-east Asia.

*Anopheles maculatus *was collected as larvae from six locations, all inland below 500 m asl. *Anopheles maculatus *appeared at the end of the wet season (from May onwards) when large numbers of larvae were commonly found in shallow pools formed in the gravel beds of receding rivers and in shallow water remaining in rice fields post harvest. *Anopheles maculatus *is one of several closely related species within the Maculatus Group [24], the members of which have recently been identified based on ITS2 sequence differences [25]. Molecular characterisation of 54 specimens (5-10 specimens from each larval site) indicated one distinguishable ITS2 RFLP genotype. Sequences of the ITS2 region from a subset of these specimens were the same (GQ500120) and matched 100% to a GenBank sequence of the same species collected from Terengganu in Malaysia and also clustered with other *An. maculatus *sequences from Malaysia.

*Anopheles sundaicus *was found at Port Hera, with eight specimens collected in HLC. The taxon *sundaicus *was recently split based on mitochondrial (COI and cyt-b) and ribosomal ITS2 markers, with the mainland type designated *Anopheles epiroticus *and the island type *An. sundaicus *[26-28]. The ITS2 sequence of the Port Hera specimens matched Variant II, which is one of four distinct ITS2 sequence variants for *An. sundaicus *[18]. Variant II occurs in Sumatra and Java (Indonesia) and can occur together with Variants I and III within a single mosquito. Variant IV appears unique to the Malaysian Borneo populations. On comparison of the cyt-b sequences of the Timor specimens to published sequences used to develop a PCR diagnostic separating *An. epiroticus, An. sundaicus *s.s., and *An. sundaicus *species E [18], we found polymorphisms that would not identify the Timor specimens as either taxa. However, phylogenetic evolutionary analysis using cyt-b sequences with published *An. sundaicus *sequences from south-east Asian individuals grouped these specimens with *An. sundaicus *individuals from Malaysia-Borneo.

*Anopheles vagus *was the most common species in the survey region with 1315 specimens identified by morphology. It was taken in larval collections, HLC and CO2 baited light traps from 32 of the 36 collection sites. It had the widest distribution of any species being collected on the coast, inland plains, and highland regions. The presence of a small pale patch of scales on the apex of the labium - a character used to separate *An. vagus *var. *vagus *from *An. vagus *var. *limosus *[29] and from *An. subpictus *[6,14] - was found in 27 of the 1315 specimens collected; the remainder had an all-black labium. Papal morphology, also used to separate *An. vagus *and *An. subpictus *[6,14,15], was not definitive in these specimens and was difficult to apply as specimens shared characters of both species. It could not be discounted that some of this material morphologically resembled *An. subpictus*.

**Figure 2:**
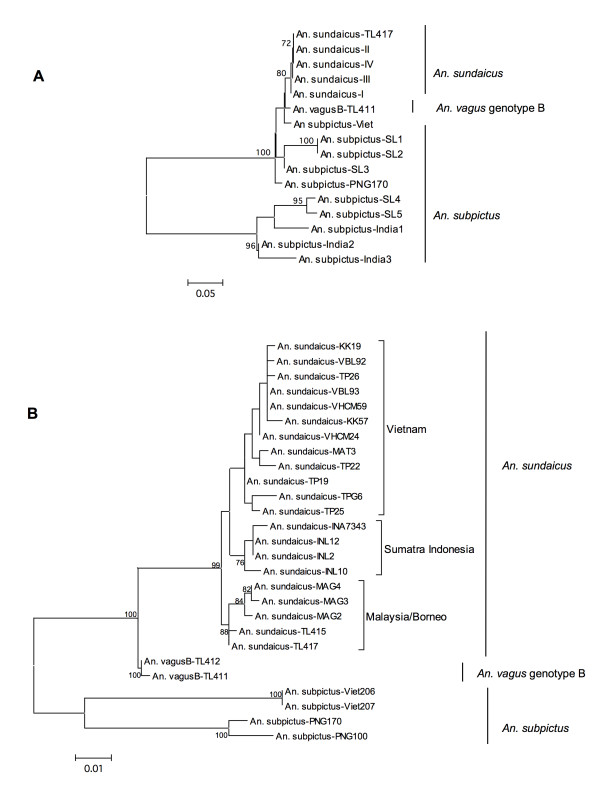
**Maximum likelihood phylogenetic trees of *An. sundaicus, An. subpictus*, and *An. vagus *genotype B generated from DNA sequences of the rDNA ITS2 (Panel A) and mtDNA cyt-b (PanelB)**. In Panel A, the ITS2 tree places *An. sundaicus *from Timor-Leste (TL417) with the *An. sundaicus *ITS2 sequence variants I-IV. However, the placement of *An. vagus *genotype B (TL411) specimens basal to the *An. sundaicus *clade suggest a more recent evolutionary connection to *An. sundaicus *as the *An. subpictus *individuals from Papua New Guinea, Indonesia, Vietnam and Sri Lanka appear paraphyletic suggesting several lineages or cryptic species exist within this morphotaxa. In Panel B the Maximum likelihood analysis of cyt-b sequences places *An. sundaicus *individuals from Timor-Leste (TL415 and TL 417) within other *An. sundaicus *species forming a well supported and separate branch with individuals from Malaysia/Borneo. The cyt-b analysis also places *An. vagus *genotype B (TL411 and TL412) basal to the *An. sundaicus *clade and paraphyletic to *An. subpictus *individuals, also indicating closer genetic affinities to *An. sundaicus *and not *An. subpictus*. For each of the trees only bootstrap branch support values over 70% are displayed.

All specimens morphologically identified as *An. vagus *were amplified and digested with the restriction enzymes *Msp *I and *Rsa *I. Both enzymes generated two unique RFLP profiles, and these genotypes were designated A (901 specimens) and B (414 specimens). The size of the undigested ITS2 differed between the two genotypes with A being approximately 700 bp and B 600 bp. All 27 specimens with white scaling on the apex of the proboscis were identified as genotype A. The ITS2 sequences of these two genotypes were compared to existing sequences in GenBank. Genotype A matched an existing sequence of *An. vagus *(FJ654649), whereas genotype B showed highest identity (98%) to *An. sundaicus *carrying the ITS2 sequence Variant I (AY768540) [18]. A neotype has recently been raised for *An. sundaicus *and a detailed morphological description provided; the neotype and associated topotypic specimens were collected from Sarawak [28]. The specimens of genotype B collected from Timor-Leste are morphologically distinct from the *An. sundaicus *neotype in that the femur and tibia are not speckled or mottled with white scales. This characteristic is considered to be of major importance in separating *An. sundaicus *from *An. vagus *and *An. subpictus *[6,14], thus suggesting here that genotype B is morphologically closer to *An. vagus *and *An. subpictus *than *An. sundaicus*. Phylogenetic assessment using: the four ITS2 sequence variants of *An. sundaicus, An. vagus *genotype B, and *An. subpictus *from PNG, Indonesia, Sri Lanka and Vietnam, strongly supports genotype B clustering with *An. sundaicus *species (Figure 2A). Additionally, assessment of the cyt-b sequence (Figure 2B) places *An. sundaicus *from Timor with *An. sundaicus *individuals collected from Malaysia and Borneo, whereas genotype B forms a separate clade basal to the *An. sundaicus *species and also separate to *An. subpictus *species from Vietnam and PNG. It is thus considered that genotype A is *An. vagus *and genotype B - provisionally termed *An. vagus *genotype B - is an unknown species resembling *An. vagus *and *An. subpictus *morphologically but showing strong genetic affinities to the *An. sundaicus *complex. The placement of genotype B closer to *An. sundaicus *than to *An. subpictus *suggests that a large cryptic species group exists within the *An. subpictus *taxon and would require a more thorough study of *An. subpictus *throughout its distribution in the Oriental and Australian Regions to further resolve this issue.

Of the 901 specimens of *An. vagus *collected only 61 were in HLC and these were all from inland sites. The larvae were taken both on the coast and inland and were found in a wide variety of water bodies including swamps (fresh and brackish), rice fields, smaller ground pools with both clay and gravel substrates, animal wallows and hoof prints, wheel ruts, concrete drains, domestic waste water polluted with detergents, truck tyres and folds in plastic wraps where it cohabited with the container breeder *Aedes albopictus*.

Specimens of *An. vagus *genotype B were commonly collected in HLC (335/414). All specimens collected by this method were from coastal sites except for one inland location (site 22, Tonobibi). *Anopheles vagus *genotype B was only collected as larvae on the coast and was more conservative in its larval habits than *An. vagus*, being found in large, well-established bodies of water including coastal lagoons (fresh and brackish) and large pools in creek lines.

### Human biting and sporozoite infections

The landing rate for all anophelines in coastal areas was 24.0 anophelines/human/night (based on 132 hours of collecting) and for inland areas 14.64 anophelines/human/night (based on 108 hours of collecting). *Anopheles barbirostris *and *An. vagus *genotype B made up 82.1% of the HLC. The landing rate of *An. vagus *genotype B was 15.6/human/night on the coast, while the rate for *An. barbirostris *was 7.44/human/night on the coast and 5.52/human/night inland.

For all species there appeared to be an observable peak feeding period early in the evening. On the coast, 24.6% of all feeding occurred in the first hour of the night from 1900 to 2000 hr, and inland, 49.3% of feeding occurred at this time. The anophelines/human/hr for 1900-2000 hr was 3.46 for all species while the average hourly landing rate after this was 1.2 anophelines/human/hr.

From the HLC 907 specimens were processed for circumsporozoite antigen, these consisted of 390 *An. barbirostris*, 61 *An. vagus*, 355 *An. vagus *genotype B, 66 *An. peditaeniatus*, 20 *An. aconitus*, 8 *An. sundaicus*, and 7 *An. annularis*. Of these, one *An. barbirostris*, collected from the inland village of Marko (site 32), was found positive for CS protein for both *Plasmodium falciparum *and *Plasmodium vivax *(210 variant); and one *An. vagus *genotype B, collected from the village of Aidabelatan (site 8), was found positive for *P. vivax *(247 variant) CS protein.

## Discussion

The data presented here on anopheline species' composition supports the concept that the anopheline fauna of Timor-Leste is of Oriental origin and contains no species from the Australian Region. Thus Timor-Leste is one of the most south-easterly limits of an Oriental anopheline fauna that has filtered down from south-east Asia over the last 5 M years.

Lien and others [9] list 14 species of *Anopheles *in Timor-Leste, this being a compilation of the studies of various earlier workers. More comprehensive studies, conducted by Portuguese workers, collected both larvae and adults from 17 locations over a period of five months, covered most of the country, and identified nine species of *Anopheles *[7,8]. The present survey covered only the Bobonaro District and the Dili area, but included all the main landforms - coastal, inland plains and highland regions - and with regards to human landing collections and larval habitats, the sampling was quite intensive. Nine species were collected in the present survey, seven of which were also collected by Fraga de Azevedo and others [7] these included: *An. barbirostris, An. aconitus, An. annularis, An. flavirostris, An. maculatus, An. sundaicus *and *An. vagus*. In addition, we collected *An. peditaeniatus *and *An. vagus *genotype B whereas Fraga de Azevedo and others [7] also collected *An. subpictus *and *Anopheles tesselatus*. Other Portuguese workers [8] added *An. nigerrimus *and *Anopheles barbumbrosus *to the records, though for Timor-Leste this latter species is more likely to be *An. barbirostris *[13].

All previous surveys on the anopheline fauna of Timor-Leste predate the advent of molecular genetic technologies (PCR and DNA sequencing) and have relied solely on morphological identification. Morphology is still the mostly commonly used method of identifying anophelines in malarious countries throughout the world. It has the benefits of being relatively inexpensive and quick (when compared to molecular methods) and can be carried out in the field. However, the variation of morphological characteristics within and between species, the practical limitations of local keys, and the presence of cryptic species within many of the *Anopheles *taxa undermines the effectiveness of this identification method. Still, preliminary morphological identification remains indispensable for initially assigning specimens to species groups and complexes, thus simplifying the task of subsequent molecular analysis.

Species identification is paramount to understanding malaria epidemiology and so the genetic characterisation of cryptic species and the subsequent use of molecular diagnostic tools provide vital support for studies into mosquito surveillance and malaria control initiatives. In this study, molecular genetic methods separated *An. flavirostris *from *An. aconitus *and differentiated *An. sundaicus *from *An. epiroticus*, supporting the concept of island and mainland species [27] with *An. sundaicus *confined to the islands of south-east Asia. Earlier workers have identified *An. argyropus, An. nigerrimus *and *An. sinensis *from the Hyrcanus Group in Timor-Leste [9]. There are problems with the morphological identification of these and other members of this group of mosquitoes throughout south-east Asia and China [30,31]. It has been suggested that there is too much variation in many of the characters commonly used to assign affinities between members of this group [31]. In the material collected here from Timor-Leste, it was difficult to morphologically separate *An. argyropus, An. nigerrimus *and *An. peditaeniatus*, however PCR and sequencing of the ITS2 confirmed all the material as *An. peditaeniatus *and it is possible that earlier collections of *An. argyropus, An. nigerrimus *and *An. sinensis *were misidentifications of this species. Several members of the Hyrcanus Group are found in Indonesia, but only *An. peditaeniatus *has been found as far east as the island of Lombok (900 km west of Timor) [32].

*Anopheles vagus *genotype B morphologically resembles *An. subpictus *and *An. vagus*. At the molecular genetic level however, this mosquito was placed, with strong branch support by the ITS2 and cyt-b markers, between *An. subpictus *and *An. sundaicus*, with closer genetic affinities to *An. sundaicus. Anopheles vagus *genotype B is probably an undescribed new species that has in the past been misidentified as *An. subpictus *and thus may be an important vector of malaria in Timor-Leste [7-9]. Like *An. sundaicus, An. subpictus *is also a species complex, but only a few ITS2 sequences were available for comparative analyses. Some of these were from India and Sri Lanka and appeared to be distantly related. To provide additional comparative data, sequences of *An. subpictus *specimens from PNG and Vietnam were included in the analysis; however, *An. vagus *genotype B could still not be placed confidently with either *An. subpictus *or *An. sundaicus*.

Interestingly, both *An. sundaicus *and *An. vagus *genotype B from Timor-Leste cannot be amplified with the *An. sundaicus *PCR diagnostic that separates *An. epiroticus, An. sundaicus *s.s, and *An. sundaicus *E [18], because they do not contain the species-specific sequence mutations required for allele-specific primer binding. Nonetheless, phylogenetically *An. sundaicus *from Timor groups with individuals from Malaysia-Borneo, which are regarded as *An. sundaicus *s.s., and not Sumatra and Java (Indonesia) individuals, which are geographically closer and regarded as *An. sundaicus *E [10]. The placement of *An. vagus *genotype B at the base of the *An. sundaicus *complex, and as sister taxa to *An. subpictus *species, supports the concept that this is a new as yet undescribed malaria vector species.

A shared evolutionary trait exists within both the *An. sundaicus *complex and the *An. subpictus *complex in that they are both coastal species that have adapted to utilising brackish water for oviposition and the development of the immature stages [10]. In this study, *An. vagus *genotype B was mainly found on the coast and the larvae of this species were collected from brackish water sites. Only one inland site was found and the ITS2 sequences of specimens from this site matched those of *An. vagus *genotype B collected from the coast; earlier workers collected specimens of *An. subpictus *from two inland locations up to 300 m asl [7]. A further detailed study utilizing both morphological and molecular characters needs to be conducted on the *An. vagus *and *An. subpictus *groups to validate existing morphological markers. For example, how important is the apical patch of pale scales on the proboscis in separating *An. vagus *and *An. subpictus*?

*Anopheles vagus*, identified in this study by morphology and confirmed by PCR-RFLP and DNA sequencing, fits the behavioural characteristics of this species in that it is a highly adaptable species, capable of utilising a wide variety of aquatic habitats including artificial containers. Its reported zoophilic behaviour [14] is supported here with only 6.7% (61/901) of specimens collected by human landing despite an abundance of larvae being found throughout the survey area.

In April-May 2001 the malaria prevalence rate was determined by mass blood surveys in seven villages located in the area where the vector surveys were conducted [1]. The parasite rate ranged from 0% at Bobonaro in the highlands to 35.3% at Batugade on the coast and on the inland plains it ranged from 6.0% at Marko to 9.0% at Maliana; these figures indicate that there was active transmission occurring in the area at the time of the vector surveys. In prior studies only *An. subpictus *has been identified with sporozoites whereas oocysts have been found in *An. barbirostris, An. sundaicus*, and *An. aconitus *[7-9]. This present study confirms the vector status of *An. barbirostris *and incriminates *An. vagus *genotype B, which is likely to be *An. subpictus *of earlier authors.

The peak feeding time for all species of *Anopheles *collected in Timor-Leste was early in the evening (1900-2000 hr). This may have implications for malaria control strategies such as indoor residual spraying or long-lasting insecticide-treated bed nets, as early night feeding vectors can avoid these measures by feeding early when most of the local population is outdoors and unprotected.

All species collected in this survey, except *An. maculatus*, were found to feed on humans and so could be potential vectors of malaria. How important this role might be will depend on their ability to achieve appreciable numbers, their host preference and their longevity. *Anopheles vagus *was very common in the survey region but appeared to be only an indifferent feeder on humans and probably plays no role in malaria transmission, confirming similar observations made by other workers [7,14]. The ability to achieve appreciable numbers will vary throughout the year depending on seasonal weather conditions producing suitable larval habitats. *Anopheles sundaicus *has been incriminated as a vector in Timor-Leste, but at the time of our surveys its numbers were very low. Higher numbers have been collected between March and November by other workers, with some of these specimens positive for oocysts [7]. Similarly, the larvae of *An. maculatus *only appeared in large numbers at the end of the survey period when the aquatic habitats it appears to favour were plentiful and so in these surveys the peak period for this species may have been missed. This highlights the limitation of surveys conducted over one season; only longitudinal surveys over several years can help to resolve seasonal fluctuations in species densities.

## Conclusions

The anopheline fauna of Timor-Leste consists of Oriental species with no species from the Australian Region. Accurate morphological identification is difficult due to the presence of cryptic taxa with overlapping morphological characters, possibly the result of genetic drift in small isolated island populations following founder events leading to morphological changes. However, the simplicity and timeliness of identification by morphology makes it invaluable in the field even if only as an initial screening tool prior to molecular analysis.

This study identified seven species of *Anopheles *morphologically; molecular methods confirmed these and in addition identified *An. flavirostris *and a new species referred to here as *An. vagus *genotype B. *Anopheles barbirostris *and *An. vagus *genotype B were the two taxa most commonly attracted to humans and both these species were found positive for *Plasmodium *CS protein.

## Competing interests

The authors declare that they have no competing interests.

## Authors' contributions

RDC designed the study, organised the field work, participated in the field collections, performed the preliminary identification using conventional PCR-RFLP and wrote the manuscript. MDE participated in the field collections and was involved in drafting and revising the manuscript. SPF organised parts of the field work, participated in the field collections and was involved in drafting and revising the manuscript. NWB was responsible for the overall molecular analysis of the specimens and was involved in drafting and revising the manuscript. All authors read and approved the final manuscript

## References

[B1] Bragonier R Reyburn H Nasveld P Edstein M Auliffe A Rainy- season prevalence of malaria in Bobonaro district, East Timor Ann Trop Med Parasitol 2002 96 739 743 10.1179/000349802125008143 12537636

[B2] David HL Some features of malaria in Dili, Portuguese Timor, during 1963-64 Ann Trop Med Parasitol 1965 59 147 152 14345277 10.1080/00034983.1965.11686292

[B3] Audley-Charles MG Whitmore TC Geological history of the region of Wallace's Line 1981 Oxford: Clarendon Press 24 35

[B4] Lee DJ Woodhill AR The anopheline mosquitoes of the Australasian Region 1944 Glebe, Sydney: Aust Med Pub Co Ltd

[B5] Brug SL Bonne-Wepster J The geographical distribution of the mosquitoes of the Malay Archipelago Chronica Natura 1947 103 179 196

[B6] Bonne-Wepster J Swellengrebel NH The anopheline mosquitoes of the Indo-Australian region 1953 Amsterdam, Netherlands: J. H. de Bussy

[B7] Fraga de Azevedo J Gandara AF Ferreira AP Contribution to the knowledge of the *Anopheles *of Portugese Timor as Vectors of Plasmodia Proceedings of the 10th International Congress of Entomology: 17-25 August 1956; Montreal, Canada

[B8] Ferreira AP Breda AVMA Estudos sobre a endemia malarica em Timor, com vista a establecerse um plano de luta contra a mesma. 3. Inquerito entomologico Anais Inst Med Trop Lisbon 1961 18 201 225

[B9] Lien JC Atmosoedjono S Usfinit AU Gundelfinger BF Observations on natural plasmodial infections in mosquitoes and a brief survey of mosquito fauna in Belu Regency, Indonesia Timor J Med Entomol 1975 12 333 337 241851 10.1093/jmedent/12.3.333

[B10] Manguin S Garros C Dusfour I Harbach RE Coosemans M Bionomics, taxonomy, and distribution of the major malaria vector taxa of *Anopheles *subgenus Cellia in Southeast Asia: An updated review Infect Genet Evol 2008 8 489 503 10.1016/j.meegid.2007.11.004 18178531

[B11] Beebe NW Saul A Discrimination of all members of the *Anopheles punctulatus *complex by polymerase chain reaction-restriction fragment length polymorphism analysis Am J Trop Med Hyg 1995 53 478 481 7485705 10.4269/ajtmh.1995.53.478

[B12] Kitchener SJ Auliff AM Rieckmann KH Malaria in the Australian Defence Force during and after participation in the International Force in East Timor (INTERFET) Med J Aust 2000 173 583 585 11379495 10.5694/j.1326-5377.2000.tb139349.x

[B13] Rohe DL Fall R A miniature battery powered CO2 baited light trap for mosquito borne encephalitis surveillance Bull Soc Vector Ecology 1979 4 24 27

[B14] Reid JA Anopheline mosquitoes of Malaya and Borneo Stud Inst Med Res Malaya 1968 31 1 520

[B15] O'Connor CT Soepanto A Illustrated key to female anophelines of Indonesia 1979 Directorate of Communicable Disease: Ministry of Health, Jakarta, Indonesia

[B16] Van Bortel W Trung HD Roelants P Harbach RE Backeljau T Coosemans M Molecular identification of *Anopheles minimus *s.l. beyond distinguishing the members of the species complex Insect Mol Biol 2000 9 335 340 10.1046/j.1365-2583.2000.00192.x 10886418

[B17] Beebe NW Maung J Hurk AF van den Ellis JT Cooper RD Ribosomal DNA spacer genotypes of the *Anopheles bancroftii *group (Diptera: Culicidae) from Australia and Papua New Guinea Insect Mol Biol 2001 10 407 413 10.1046/j.0962-1075.2001.00278.x 11881804

[B18] Dusfour I Blondeau J Harbach RE Vythilingham I Baimai V Trung HD Sochanta T Bangs MJ Manguin S Polymerase chain reaction identification of three members of the *Anopheles sundaicus *(Diptera: Culicidae) complex, malaria vectors in Southeast Asia J Med Entomol 2007 44 723 31 10.1603/0022-2585(2007)44[723:PCRIOT]2.0.CO;2 17915501

[B19] Thompson JD Higgins DG Gibson TJ CLUSTAL W: improving the sensitivity of progressive multiple sequence alignment through sequence weighting, position specific gap penalties and weight matrix choice Nucleic Acids Research 1994 22 4673 4680 10.1093/nar/22.22.4673 7984417 PMC308517

[B20] Guindon S Gascuel O A simple, fast, and accurate algorithm to estimate large phylogenies by maximum likelihood Syst Biol 2003 52 696 704 10.1080/10635150390235520 14530136

[B21] Ryan JR Davé K Collins KM Hochberg L Sattabongkot J Coleman RE Dunton RF Bangs MJ Mbogo CM Cooper RD Schoeler GB Rubio Y Magris M Romero LI Padilla N Quakyi IA Leke RG Akinpelu O Evans B Walsey M Patterson P Wirtz RA Chan AST Extensive multiple test center evaluation of the VecTest malaria antigen panel assay Med Vet Entomol 2002 16 321 327 10.1046/j.1365-2915.2002.00368.x 12243234

[B22] Beier JC Asiago CM Onyango FK Koros JK ELISA absorbance cut-off method affects malaria sporozoite rate determination in wild Afrotropical *Anopheles* Med Vet Entomol 1988 2 259 264 10.1111/j.1365-2915.1988.tb00193.x 2980182

[B23] Ma Y Xu J The Hyrcanus Group of *Anopheles *(*Anopheles*) in China (Diptera: Culicidae): Species discrimination and phylogenetic relationships inferred by ribosomal DNA internal transcribed spacer 2 sequences J Med Entomol 2005 42 610 619 10.1603/0022-2585(2005)042[0610:THGOAA]2.0.CO;2 16119550

[B24] Rattanarithikul R Green CA Formal recognition of the species of the *Anopheles maculatus *group (Diptera: Culicidae) occurring in Thailand, including the descriptions of two new species and a preliminary key to females Mosq Syst 1986 18 246 278

[B25] Walton C Somboon P O'Loughlin SM Zhang S Harbach RE Linton YM Chen B Nolan K Duong S Fong MY Vythilingum I Mohammed ZD Trung HD Butlin RK Genetic diversity and molecular identification of mosquito species in the *Anopheles maculatus *group using the ITS2 region of rDNA Infect Genet Evol 2006 7 93 102 10.1016/j.meegid.2006.05.001 16782411

[B26] Dusfour I Linton YM Cohuet A Harbach RE Baimai V Trung HD Chang MS Matusop A Manguin S Molecular evidence of speciation between island and continental populations of *Anopheles (Cellia) sundaicus *(Diptera: Culicidae), a principal malaria vector taxon in Southeast Asia J Med Entomol 2004 41 287 295 10.1603/0022-2585-41.3.287 15185927

[B27] Linton YM Dusfour I Howard TM Ruiz LF Duc Manh N Ho Dinh T Sochanta T Coosemans M Harbach RE *Anopheles (Cellia) epiroticus *(Diptera: Culicidae), a new malaria vector species in the Southeast Asia Sundaicus Complex Bull Entomol Res 2005 95 329 339 10.1079/BER2005364 16048681

[B28] Linton YM Harbach RE Seng CM Anthony TG Matusop A Morphological and molecular identity of *Anopheles (Cellia) sundaicus *(Diptera: Culicidae), the nominotypical member of a malaria vector species complex in Southeast Asia Syst Entomol 2001 26 357 366 10.1046/j.1365-3113.2001.00153.x

[B29] Colless DH The anopheline mosquitoes of north-west Borneo Proc Linn Soc NSW 1948 73 71 119

[B30] Reid JA The *Anopheles hyrcanus *Group in South-East Asia (Diptera: Culicidae) Bull Entomol Res 1953 44 5 76 10.1017/S0007485300022938

[B31] Harrison BA Southeast Asia Mosquito Project A new interpretation of affinities within the *Anopheles hyrcanus *Complex of Southeast Asia Mosq Syst 1972 4 73 83

[B32] O'Connor CT The *Anopheles hyrcanus *Group in Indonesia Mosq Syst 1980 12 293 305

